# Effect of a smartphone application on dispatcher-assisted CPR and AED response in simulated out-of-hospital cardiac arrest

**DOI:** 10.1016/j.resplu.2026.101471

**Published:** 2026-08-31

**Authors:** Duangpon Thepmanee, Pramote Papukdee, Rossakorn Klaiangthong

**Affiliations:** Department of Disaster and Emergency Medical Operation, Faculty of Science and Health Technology, Navamindradhiraj University, Bangkok, Thailand

**Keywords:** Simulation, Simulation-based research, Out-of-hospital cardiac arrest, Dispatcher-assisted CPR, AED

## Abstract

**Objective:**

Early bystander cardiopulmonary resuscitation (CPR) and rapid automated external defibrillator (AED) use are key determinants of survival following out-of-hospital cardiac arrest (OHCA). Smartphone applications may enhance dispatcher-assisted CPR (DA-CPR) and facilitate early AED access; however, evidence regarding their effectiveness in standardized simulation settings remains limited. This study evaluated the effectiveness of a smartphone application for DA-CPR and AED response during simulated OHCA.

**Methods:**

This randomized simulation-based study was conducted using standardized OHCA scenarios in a controlled simulation environment. Fifty emergency medical dispatch personnel and 190 trained lay responders were randomly assigned to either a smartphone application group or a standard practice group. Primary outcomes were time to CPR initiation and time to first AED shock. Secondary outcomes included time to OHCA recognition, time to scenario completion, and high-quality CPR performance. Outcomes were compared using independent t-tests or Welch’s t-tests, as appropriate.

**Results:**

Among dispatchers (*n* = 50), smartphone application use was associated with a shorter time to CPR initiation than the control condition (mean difference [MD], −0.56 min; 95% confidence interval [CI], −1.05 to −0.07; *p* = 0.026). Among lay responders (*n* = 190), application use was associated with shorter times to OHCA recognition (MD, −0.13 min; 95% CI, −0.21 to −0.05; *p* = 0.001) and scenario completion (MD, −0.24 min; 95% CI, −0.42 to −0.06; *p* = 0.011), but a longer time to CPR initiation (MD, 0.24 min; 95% CI, 0.01–0.47; *p* = 0.038). No statistically significant between-group differences were observed in the time to first AED shock or overall high-quality CPR performance.

**Conclusion:**

In standardized simulated OHCA scenarios, use of the MySOS app was associated with a shorter time to first compression among dispatchers but a longer time to first compression among laypersons. No significant association was observed between MySOS use and time to first shock.

## Introduction

Out-of-hospital cardiac arrest (OHCA) remains a major global public health challenge, with an estimated incidence of 50–60 cases per 100,000 person-years and overall survival rates below 10% in many countries, including Thailand.^1^, ^2^ Survival after OHCA is highly time-dependent and relies on prompt initiation of cardiopulmonary resuscitation (CPR) and early defibrillation with an automated external defibrillator (AED) before emergency medical services (EMS) arrival. When initiated within the first 3–5 min after collapse, these interventions substantially improve survival and neurological outcomes.^3^, ^4^

Bystander CPR (BCPR) is among the strongest predictors of survival following OHCA, increasing survival by up to 31% when initiated before EMS arrival.^5^, ^6^ Early AED use combined with timely BCPR further improves survival and favorable neurological outcomes.^7^ Nevertheless, rates of BCPR and public AED use remain suboptimal in many settings, particularly in low- and middle-income countries, highlighting the need for strategies that strengthen early community response.

Smartphone technology has emerged as a promising strategy for strengthening the early links in the chain of survival by facilitating dispatcher-assisted CPR (DA-CPR), alerting nearby responders, and directing users to the nearest AED. Previous studies have suggested that smartphone-based systems may improve response processes and reduce defibrillation delays.^8^, ^9^, ^10^, ^11^ However, evidence regarding their effect on communication between emergency medical dispatch personnel and lay responders, as well as other time-critical resuscitation processes, remains limited. Furthermore, evaluating these processes during real OHCA is challenging because patient characteristics, scene conditions, and AED accessibility vary considerably and cannot be standardized.

Simulation-based research provides a controlled and reproducible environment in which standardized OHCA scenarios, identical dispatcher information, consistent AED placement, and uniform outcome assessment can be achieved while avoiding the ethical and practical limitations of real cardiac arrest research. In Thailand, a smartphone application has been developed to support emergency medical dispatch personnel and lay responders during OHCA events^12^; however, its effectiveness in facilitating DA-CPR and AED response has not been systematically evaluated. Therefore, this randomized simulation-based study aimed to evaluate the effectiveness of a smartphone application in improving time to CPR initiation and time to first AED shock during standardized simulated OHCA scenarios in Bangkok, Thailand.

## Methods

### Study design

This randomized simulation-based experimental study evaluated the effectiveness of a smartphone application for DA-CPR and AED response using standardized OHCA scenarios. Simulation sessions were conducted in Bangkok, Thailand, on August 9–10, 2022, for emergency medical dispatch personnel and on August 24, 2022, for trained lay responders.

Simulation was used as an investigational methodology because it allowed standardized OHCA scenarios, identical dispatcher information, consistent AED placement, and reproducible assessment of time-critical resuscitation processes that cannot be ethically or practically standardized during real OHCA events. Because no patients or clinical interventions were involved, the study was not considered a clinical trial and was therefore not prospectively registered.

### Participants

Participants included emergency medical dispatch personnel from the Bangkok Emergency Medical Center (Erawan Center), comprising call takers, nurses, paramedics, and emergency medical technicians who routinely performed emergency medical dispatch duties, together with trained lay responders from Suan Dusit University. Because the dispatcher and lay responder groups were evaluated independently, the simulation sessions were conducted on separate dates. During the dispatcher simulation sessions, the lay responder role was performed by simulation volunteers who had previously completed standardized training in emergency call activation, chest-compression CPR, and AED use. These volunteers followed standardized simulation scripts throughout all scenarios to ensure consistency across sessions. The simulation volunteers were not study participants and were not included in the data analysis.

Lay responders were undergraduate students who were not medical students or healthcare professionals. Eligible participants were aged 18 years or older and had completed standardized Basic Life Support (BLS) an AED training before enrolment. Emergency medical dispatch personnel performed their routine dispatch duties during the simulations, whereas trained lay responders acted as simulated first responders at the scene. Written informed consent was obtained from all study participants before participation.

### Sample size

No formal sample size calculation was performed because this was an exploratory simulation-based study conducted under predefined operational and logistical conditions. The sample size was therefore determined pragmatically based on the number of eligible emergency medical dispatch personnel and trained lay responders available during the scheduled simulation sessions. A total of 50 emergency medical dispatch personnel and 190 trained lay responders were enrolled and included in the final analysis.

### Simulation environment

The study was conducted in a controlled simulation environment designed to represent a standardized urban OHCA setting in Bangkok, Thailand. The simulation area covered approximately 0.4 km^2^ and included three publicly accessible AED stations located approximately 200–300 m apart to reflect realistic AED accessibility. All simulation sessions were conducted under standardized environmental conditions using identical dispatch information, participant instructions, OHCA scenarios, and AED locations. Emergency medical dispatch personnel and lay responders assigned to the intervention group were pre-registered in the smartphone application to enable real-time communication, responder activation, location tracking, and AED navigation throughout the simulation.

### Simulator

The simulated victim was represented using a Resusci Anne QCPR mannequin (Laerdal Medical, Stavanger, Norway).

### Participant orientation

All participants completed a standardized 3-hour orientation before the simulation. The orientation included instruction on the principles of BLS, AED use, and the study procedures. Participants assigned to the intervention group also received standardized training on the functions of the smartphone application, including responder activation, CPR guidance, and AED navigation.

### Smartphone application

**Fig. 1:**
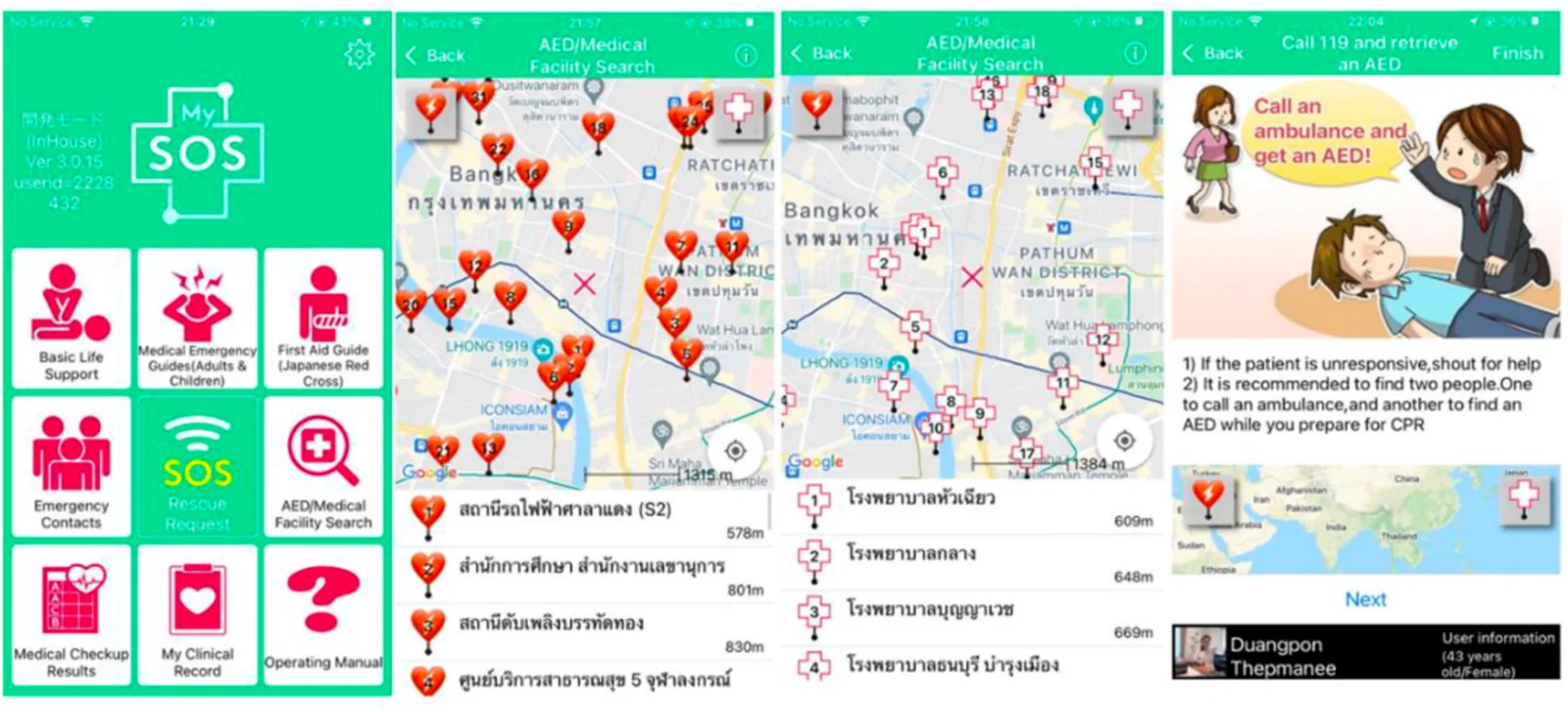
**Functional components of the smartphone application used in OHCA scenarios**.

The smartphone application was developed to support early OHCA response by facilitating DA-CPR and AED retrieval. The application's core functions included OHCA alerts, CPR guidance, responder activation, real-time location tracking, and AED location guidance. During the simulation, all participants assigned to the intervention group used smartphones preloaded with the application (Fig. 1).

### Simulation scenarios

**Fig. 2:**
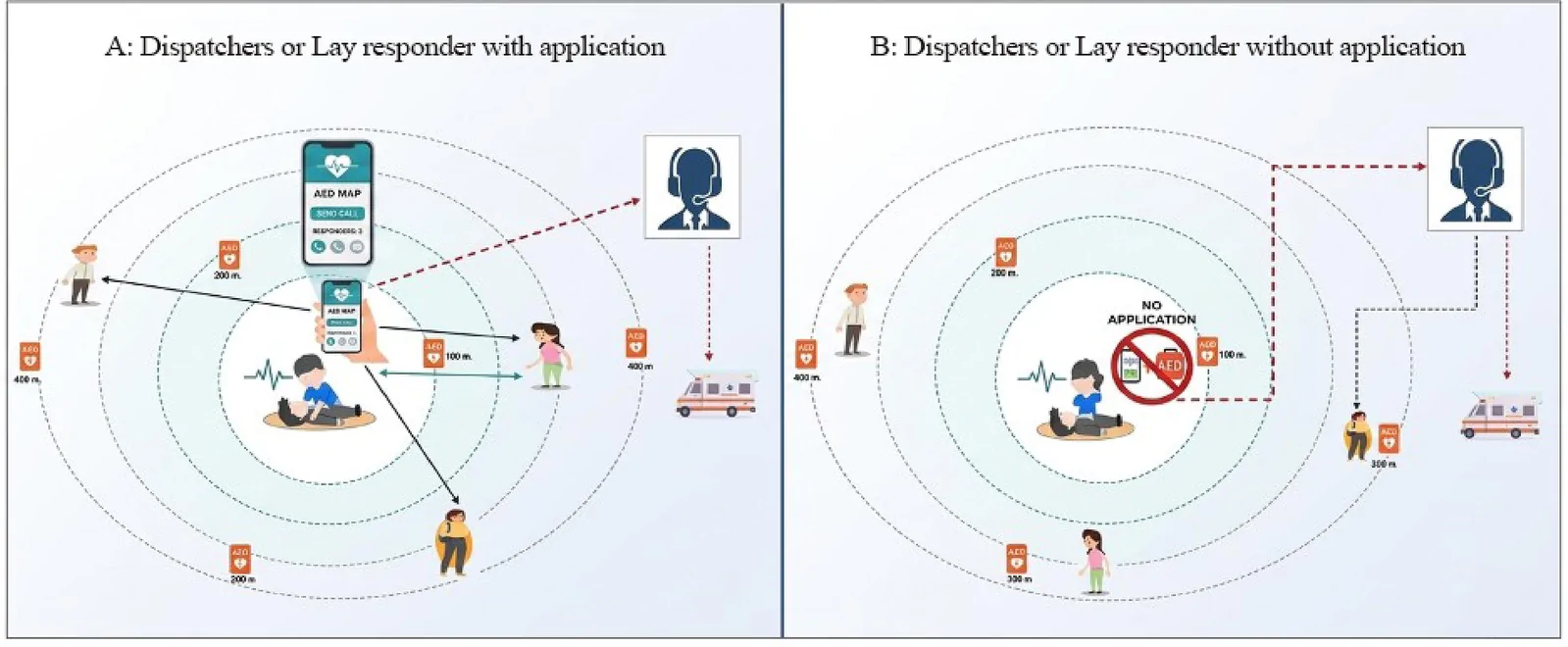
**Schematic representation of OHCA response with and without the smartphone application. (A) With application: real-time alert, AED location guidance, and responder activation. (B) Without application: standard response without digital support**.

Three standardized scripted OHCA scenarios with different AED locations were developed before participant enrolment. In each scenario, one emergency medical dispatch personnel member coordinated two lay responders. Two AEDs were positioned on the third floor of nearby buildings approximately 200–300 m from the simulated OHCA location. Scenario progression, dispatch information, AED availability, and participant instructions were identical across groups to minimize performance variability. Participants were instructed to recognize OHCA, initiate CPR, retrieve the nearest AED, and complete the simulated resuscitation as rapidly as possible (Fig. 2).

### Randomization and blinding

Emergency medical dispatch personnel and lay responders were randomized separately in a 1:1 ratio using a computer-generated random sequence. Owing to the nature of the intervention, participant blinding was not feasible. However, the primary outcomes were determined using standardized time-stamped records generated by both the smartphone application and the simulation monitoring system, thereby reducing observer bias.

### Statistical analysis

Simulation events were recorded using synchronized application time stamps and standardized simulation monitoring tools. The individual participant was the unit of analysis. Continuous variables were compared using Student's *t*-test or Welch's *t*-test when equality of variance assumptions was violated. Categorical variables were compared using the chi-square test. Two-sided *p* values < 0.05 were considered statistically significant. Statistical analyses were performed separately for emergency medical dispatch personnel and lay responders.

### Outcome measures

The primary outcomes were time from OHCA recognition to initiation of BCPR and time to first AED shock delivery. Secondary outcomes included time to scenario completion, time to OHCA recognition, and adherence to predefined high-quality CPR performance measures. Time outcomes were recorded using standardized simulation monitoring procedures and synchronized time records generated during each simulation. Two independent investigators reviewed all recordings.

### Reporting guideline

This study was reported in accordance with the Simulation-Based Research Reporting Guideline.^13^ The completed reporting checklist is provided in Appendix A (Supplementary Material).

### Ethical considerations

The study protocol was approved by the Bangkok Research Ethics Committee, Navamindradhiraj University, Thailand (Approval No. E012h/64).

## Results

### Simulation completion

**Fig. 3:**
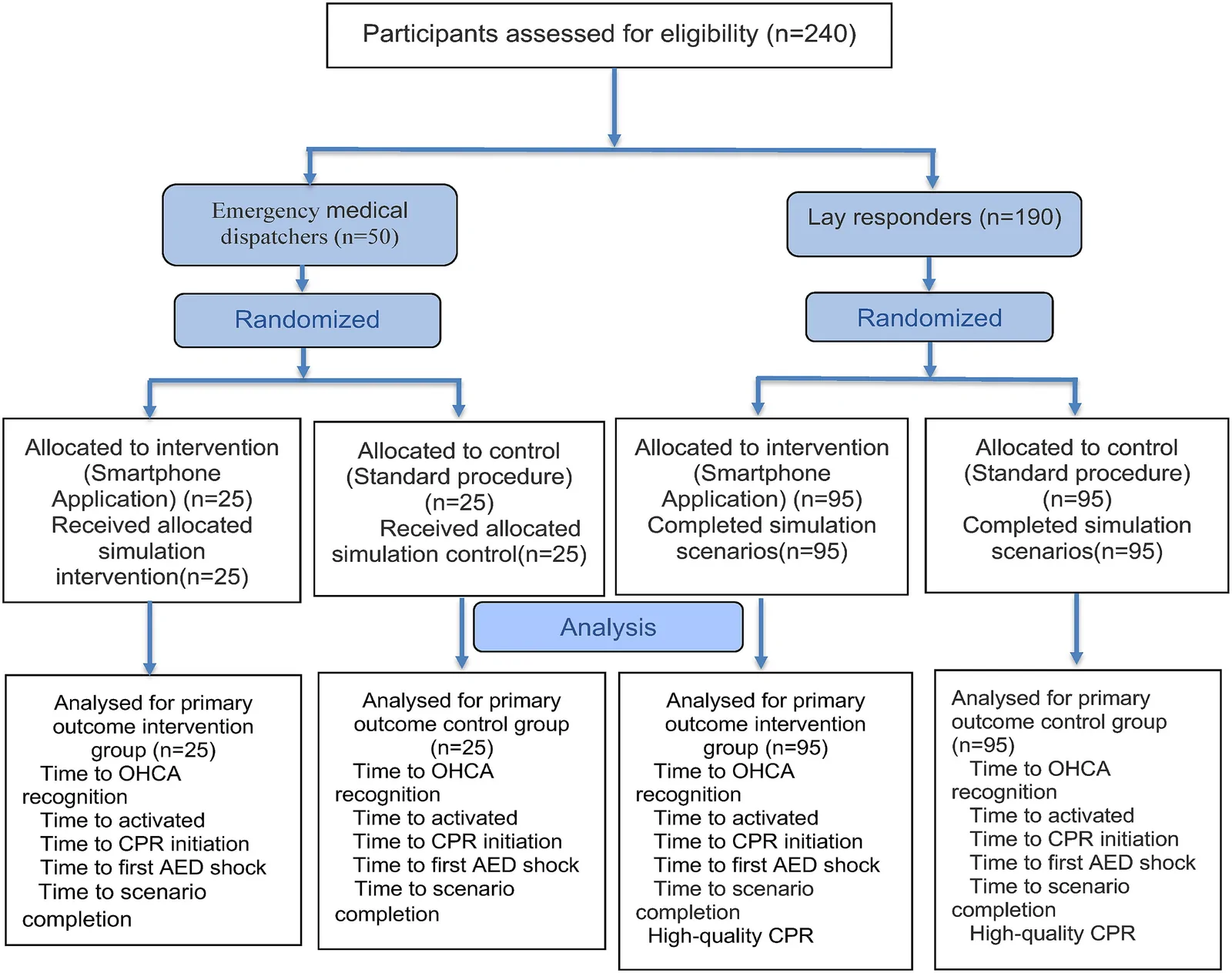
**Flow diagram of participant enrollment, randomization, and analysis in the simulation-based study**.

A total of 240 participants, including 50 emergency medical dispatch personnel and 190 trained lay responders, were randomized to the intervention or control groups. All randomized participants completed their assigned simulation scenarios and were included in the final analyses. No participants withdrew during the study, and no protocol deviations, missing outcome data, or technical failures affecting outcome assessment occurred during the simulation sessions (Fig. 3).

### Participant characteristics

A total of 50 emergency medical dispatch personnel from the Bangkok Emergency Medical Center (Erawan Center) participated in the simulation and were randomly assigned to the smartphone application group (*n* = 25) or the control group (*n* = 25). The majority of participants in the dispatch personnel group were female (*n* = 30, 60%), worked as call-takers (*n* = 28, 56%), and had 1–2 years of professional experience (*n* = 40, 80%). Among the lay responders, 190 participants were enrolled and randomly allocated to the smartphone application group (*n* = 95) or the control group (*n* = 95). Among lay responders, 134 participants (70.5%) were female and 56 (29.5%) were male. Baseline characteristics of both study populations are summarized in Table 1.

**Table 1:** Demographic characteristics of dispatcher and lay responder participants.

**Characteristic**	**Dispatcher (*n* = 50)**	**Lay responder (*n* = 190)**
**Sex**		
Female	30 (60%)	134 (70.5%)
Male	20 (40%)	56 (29.5%)
**Position**		
Call taker, *n* (%)	28 (56%)	−
Nurse, *n* (%)	1 (2%)	−
Paramedics, *n* (%)	15 (30%)	−
EMT, *n* (%)	6 (12%)	−
Students, *n* (%)	−	190 (100%)
**Experience**		
1–2 years, *n* (%)	40 (80%)	−
>2–5 years, *n* (%)	2 (4%)	−
>5–10 years, *n* (%)	3 (6%)	−
>10 years, *n* (%)	5 (10%)	−

EMT = Emergency Medical Technician.

### Primary outcomes

Among emergency medical dispatch personnel, MySOS use was associated with a shorter time to CPR initiation (MD, −0.56 min; 95% CI, −1.05 to −0.07; *p* = 0.026). No statistically significant between-group differences were observed in the time to OHCA recognition, activation, first AED shock, or scenario completion. Among lay responders, MySOS use was associated with shorter times to OHCA recognition (MD, −0.13 min; 95% CI, −0.21 to −0.05; *p* = 0.001) and scenario completion (MD, −0.24 min; 95% CI, −0.42 to −0.06; *p* = 0.011), but a longer time to CPR initiation (MD, 0.24 min; 95% CI, 0.01–0.47; *p* = 0.038). The time to first AED shock did not differ significantly between groups (MD, −0.20 min; 95% CI, −0.50 to 0.10; *p* = 0.188) (Table 2).

**Table 2:** Group comparisons of dispatcher and lay responder outcomes (min).

**Outcome**	**Test**	**MySOS, Mean (SD)**	**Control, Mean (SD)**	**Mean difference (95% CI)**	***t***	**df**	***p*-value**
**Dispatcher outcomes**		***n* = 25**	***n* = 25**				
Time to OHCA recognition	Student’s *t*-test	0.397 (0.162)	0.41 (0.107)	−0.013 (−0.091 to 0.065)	−0.33	48	0.743
Time to activation	Student’s *t*-test	1.51 (1.08)	1.89 (1.39)	−0.380 (−1.087 to 0.327)	−1.08	48	0.285
Time to CPR initiation	Welch’s *t*-test	1.39 (0.588)	1.95 (1.07)	−0.560 (−1.050 to −0.070)	−2.31	37.3	0.026*
Time to first AED shock	Student’s *t*-test	3.9 (1.7)	4.63 (1.6)	−0.730 (−1.668 to 0.208)	−1.56	48	0.125
Time to scenario completion	Student’s *t*-test	7.00 (2.23)	7.00 (2.08)	0.000 (−1.226 to 1.226)	0.00	48	1.000

**Lay responder outcomes**		***n* = 95**	***n* = 95**				
Time to OHCA recognition	Welch’s *t*-test	0.40 (0.242)	0.53 (0.300)	−0.130 (−0.208 to −0.052)	−3.28	180	0.001*
Time to activation	Student’s *t*-test	0.80 (0.564)	0.81 (0.512)	−0.010 (−0.163 to 0.143)	−0.13	188	0.897
Time to CPR initiation	Welch’s *t*-test	2.01 (0.867)	1.77 (0.708)	0.240 (0.013–0.467)	2.09	181	0.038*
Time to first AED shock	Student’s *t*-test	4.50 (1.07)	4.70 (1.02)	−0.200 (−0.499 to 0.099)	−1.32	188	0.188
Time to scenario completion	Student’s *t*-test	8.76 (0.564)	9.00 (0.719)	−0.240 (−0.424 to −0.056)	−2.56	188	0.011*

*Note.* Data are presented as mean (SD). Mean differences were calculated as MySOS minus control; negative values indicate shorter times in the MySOS group. The *t* statistics, degrees of freedom, and *p* values from the original analyses were retained. Welch’s *t*-test was used when the equal-variance assumption was not met; Student’s *t*-test was used for all other comparisons. *p* < 0.05 was considered statistically significant. CI, confidence interval; CPR, cardiopulmonary resuscitation; OHCA, out-of-hospital cardiac arrest; AED, automated external defibrillator.

* *p* < 0.05.

### Secondary outcomes

**Fig. 4:**
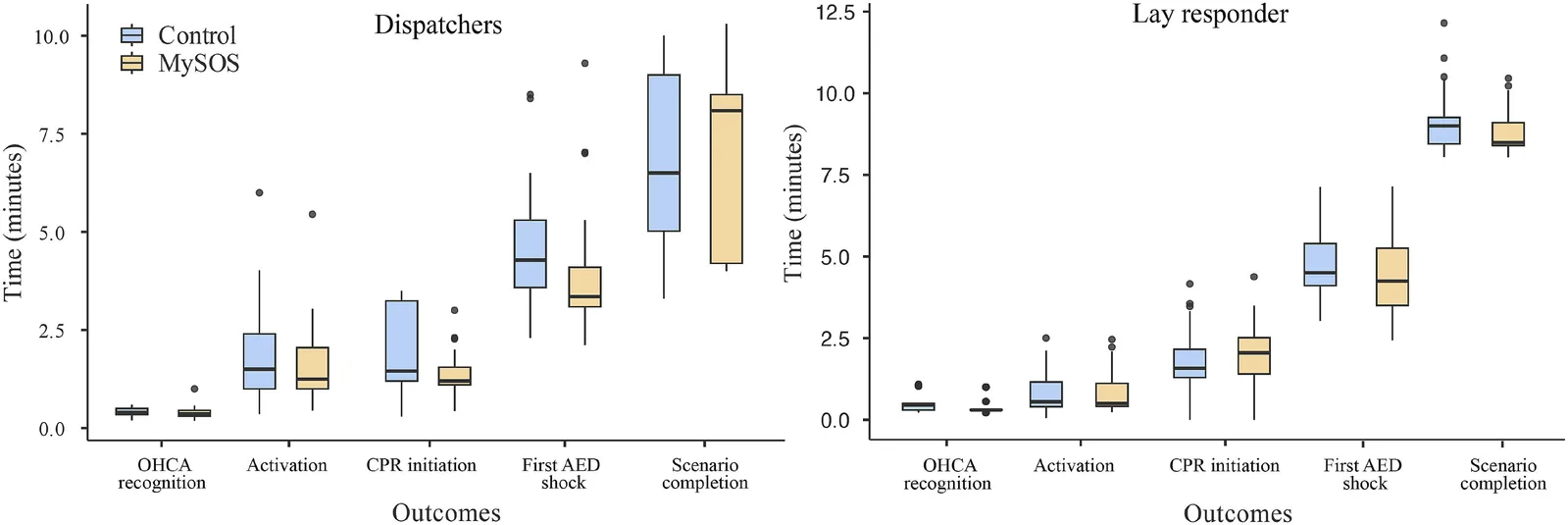
**Comparison of time-to-action outcomes between dispatchers and lay responders with and without the smartphone application**.

Fig. 4 presents the time-to-action outcomes for emergency medical dispatch personnel and lay responders. Consistent with the findings presented in Table 2, MySOS use was associated with shorter times to OHCA recognition and scenario completion among lay responders but a longer time to CPR initiation. Among emergency medical dispatch personnel, MySOS use was associated with a shorter time to CPR initiation. The time to first AED shock did not differ significantly between the MySOS and control groups in either study population.

### High-quality CPR performance

High-quality CPR performance among lay responders is summarized in Table 3. No significant differences were observed between the MySOS and control groups in chest compression quality, compression depth, or the proportion of participants achieving >75% adherence to the predefined high-quality CPR (HQ-CPR) criteria.

**Table 3:** Comparison of high-quality CPR performance between the MySOS and control groups among lay responders.

**High-quality CPR**	**MySOS (*n* = 95)**	**Control (*n* = 95)**	**Absolute difference (95% CI)**	**Effect estimate (95% CI)**	***p*-value**
Chest compression (%), median (IQR)	80 (50–100)	100 (75–100)	−	−	0.223
>75%, *n* (%)	70/95 (73.7)	71/95 (74.7)	−2.62 (−15.05 to 9.82)^a^	0.87 (0.45–1.67)^b^	0.680
Depth (%), median (IQR)	100 (75–100)	100 (75–100)	−	−	0.249
>75%, *n* (%)	73/95 (76.8)	75/95 (78.9)	−3.74 (−15.53 to 8.04)^a^	0.80 (0.40–1.60)^b^	0.534

*Abbreviation:* IQR, interquartile range.

a Absolute difference is the difference in proportions.

b Effect estimate is odds ratio (2-sided 95%CI) from a logistic model.

## Discussion

### Principal findings

Smartphone application use was associated with different time-to-action outcomes among emergency medical dispatch personnel and lay responders. Among dispatch personnel, MySOS use was associated with a shorter time to CPR initiation. In contrast, among lay responders, MySOS use was associated with a longer time to CPR initiation. No statistically significant between-group differences were detected in CPR quality or the time to first AED shock in either study population. These findings reflect the primary outcome data and suggest that the association between application use and CPR initiation may differ according to user group. Among lay responders, MySOS use was also associated with shorter times to OHCA recognition and scenario completion. These findings may indicate that smartphone-based support can facilitate certain early response processes; however, they should be interpreted cautiously because multiple outcomes were compared, increasing the possibility of type I error. The longer time to CPR initiation may partly reflect the additional cognitive and interaction demands associated with using the application, although this explanation was not directly evaluated in the present study. Previous studies have reported that smartphone-based responder systems and AED mapping or responder-activation systems may support emergency response and facilitate earlier defibrillation in real-world settings.^9^, ^10^, ^14^, ^15^ Nevertheless, MySOS use was not associated with a shorter time to first AED shock in the present standardized simulation. Overall, the findings suggest a potential trade-off between digital guidance and immediate action, particularly among lay responders. Further refinement of application usability and interface design may help minimize interaction-related delays, but this possibility requires evaluation in adequately powered real-world studies.^8^

### Comparison with previous studies

The present findings are broadly consistent with previous studies reporting that smartphone-based responder systems may support early OHCA recognition and emergency response processes.^9^, ^14^, ^15^ Similarly, MySOS use was associated with shorter times to OHCA recognition and scenario completion among lay responders. However, whereas some real-world studies reported shorter times to CPR initiation and defibrillation,^9^, ^10^, ^15^ MySOS use in the present study was associated with a longer time to CPR initiation among lay responders and was not associated with a shorter time to first AED shock. These differences may be related to the simulated study setting, participant characteristics, application design, or the additional interaction demands associated with using the application. Because cognitive load was not directly measured, its potential contribution to the observed delay should be regarded as a hypothesis requiring further investigation.

### Interpretation and clinical implications

These findings suggest that smartphone-assisted systems may support some early emergency response processes, particularly OHCA recognition. However, the longer time to CPR initiation observed among lay responders highlights the need to minimize application-related interaction during time-critical emergencies. The absence of a statistically significant difference in the time to first AED shock further indicates that improvements in recognition or scenario completion may not necessarily translate into earlier defibrillation. Given the simulated setting and the multiple outcomes examined, these findings should be interpreted cautiously and should not be considered evidence of clinical effectiveness. Future studies should evaluate the implementation, usability, and clinical effectiveness of smartphone-assisted emergency response systems in adequately powered real-world settings, with particular attention to reducing delays in the initiation of life-saving interventions.

### Strengths

This study has several strengths. First, the randomized simulation-based design allowed standardized comparisons between the MySOS and control conditions while reducing potential confounding. Second, the use of standardized OHCA scenarios, dispatcher information, participant instructions, and AED placement ensured consistency across simulation sessions. Third, objective time-to-action outcomes obtained through standardized monitoring procedures enabled consistent assessment under controlled conditions. Finally, the inclusion of both emergency medical dispatch personnel and lay responders provided insight into how associations between smartphone application use and emergency response performance may differ between user groups.

### Limitations

This study has several limitations. First, the simulations were conducted in a controlled environment with trained participants, which may limit the generalisability of the findings to real-world OHCA events involving untrained members of the public and unpredictable environmental conditions. The simulations could not fully reproduce the emotional stress, distractions, or decision-making pressures encountered during actual cardiac arrest events. Participants were also aware that they were being observed, potentially influencing their performance through the Hawthorne effect. Second, participants were assessed immediately after the orientation session. Therefore, the findings may reflect short-term performance following training, and the long-term retention of CPR skills and application-use proficiency was not evaluated. Third, AED locations and response distances were standardized across scenarios, which may have limited the ability to detect potential differences associated with application-guided AED navigation. Fourth, all lay responders were university students whose digital literacy and familiarity with smartphone technology may differ from those of the general population.

Finally, no formal a priori sample size calculation was performed, and the study may therefore have been insufficiently powered to detect small between-group differences. Multiple outcomes were examined without adjustment for multiple comparisons, increasing the risk of type I error. Accordingly, statistically significant findings, particularly those relating to outcomes other than CPR initiation and the time to first AED shock, should be regarded as exploratory. These findings should be interpreted cautiously and confirmed in adequately powered real-world studies involving more diverse participant populations.

### Generalizability

Although the simulation incorporated key components of the urban OHCA response system in Bangkok, performance under simulated conditions may differ from that during real emergencies because emotional stress, environmental complexity, patient variability, and operational constraints cannot be fully replicated. Furthermore, the lay responders were trained university students and may not represent members of the general public with different levels of CPR experience, digital literacy, or familiarity with smartphone applications. The findings may also not be directly applicable to rural areas or emergency response systems with different dispatcher protocols, AED availability, response distances, or technological infrastructure. Therefore, caution is warranted when extrapolating these findings to real-world OHCA events and other emergency medical service settings.

### Cognitive aids

The longer time to CPR initiation observed among lay responders using MySOS may be related, at least in part, to the additional interaction and information-processing demands associated with using the application during the initial response. A recent systematic review of cognitive aids in simulated resuscitation reported that, although such aids may support performance among healthcare professionals, some studies involving lay responders found less favorable resuscitation performance.^16^ Lay responders may require additional time to interpret decision support, navigate the application, and determine the appropriate action before initiating chest compressions. In the present study, the longer time to CPR initiation was not accompanied by a statistically significant between-group difference in the measured indicators of CPR quality. However, cognitive load was not directly assessed, and the study was not designed to establish whether application interaction caused the observed difference in CPR initiation time. This interpretation should therefore be considered hypothesis-generating. Further studies incorporating formal measures of cognitive workload and user interaction are needed to determine whether refinements in application usability and interface design can reduce interaction-related delays while supporting the timely initiation of CPR.

## Conclusions

In standardized simulated OHCA scenarios, smartphone application use was associated with a shorter time to first compression among dispatchers but a longer time to first compression among laypersons. No significant association was observed between smartphone application use and time to first AED shock or CPR quality.

## CRediT authorship contribution statement

**Duangpon Thepmanee:** Writing – review & editing, Writing – original draft, Visualization, Validation, Supervision, Resources, Project administration, Methodology, Investigation, Funding acquisition, Formal analysis, Data curation, Conceptualization. **Pramote Papukdee:** Writing – review & editing, Software. **Rossakorn Klaiangthong:** Writing – review & editing, Formal analysis.

## Funding

This research was supported by the National Institute for Emergency Medicine (NIEMS) and Thailand Science Research and Innovation (TSRI) under Grant No. 63/2566. Additional institutional support was provided by Navamindradhiraj University.

## Declaration of competing interest

The authors declare that they have no known competing financial interests or personal relationships that could have appeared to influence the work reported in this paper.

## Appendix A Supplementary material

Completed simulation-based research reporting checklist^13^ (https://doi.org/10.1097/SIH.0000000000000150).

## References

[1] Perkins G.D., Handley A.J., Koster R.W., Castrén M., Smyth M.A., Olasveengen T. (2018). European Resuscitation Council guidelines for resuscitation 2018: Section 2. Adult basic life support and automated external defibrillation. Resuscitation.

[2] Gräsner J.T., Lefering R., Koster R.W., Masterson S., Böttiger B.W., Herlitz J. (2016). EuReCa ONE–27 Nations, ONE Europe, ONE Registry: a prospective one month analysis of out-of-hospital cardiac arrest outcomes in 27 countries in Europe. Lancet.

[3] Holmberg M.J., Vognsen M., Andersen M.S., Donnino M.W., Andersen L.W. (2017). Bystander cardiopulmonary resuscitation and survival in out-of-hospital cardiac arrest. Circulation.

[4] Monsieurs K.G., Nolan J.P., Bossaert L.L., Greif R., Maconochie I.K., Nikolaou N.I. (2021). European Resuscitation Council guidelines for resuscitation 2021. Resuscitation.

[5] Sasson C., Rogers M.A., Dahl J., Kellermann A.L. (2010). Predictors of survival from out-of-hospital cardiac arrest: a systematic review and meta-analysis. Circulation.

[6] Iwami T., Kawamura T., Hiraide A., Berg R.A., Hayashi Y., Nishiuchi T. (2007). Effectiveness of bystander-initiated cardiac-only resuscitation for patients with out-of-hospital cardiac arrest. Resuscitation.

[7] Koster R.W., Baubin M.A., Bossaert L.L., Caballero A., Cassan P., Castrén M. (2010). European Resuscitation Council guidelines for resuscitation 2010: Section 2. Adult basic life support and use of automated external defibrillators. Lancet.

[8] Ringh M., Rosenqvist M., Hollenberg J., Jonsson M., Fredman D., Nordberg P. (2015). Mobile-phone dispatch of laypersons for CPR in out-of-hospital cardiac arrest. JAMA.

[9] Andelius L., Malta Hansen C., Lippert F.K., Karlsson L., Torp-Pedersen C., Kjær Ersbøll A. (2019). Smartphone activation of citizen responders to facilitate defibrillation in out-of-hospital cardiac arrest. Circulation.

[10] Zijlstra J.A., Stieglis R., Riedijk F., Smeekes M., van der Worp W.E., Koster R.W. (2020). Local lay rescuers with AEDs, alerted by text messages, contribute to early defibrillation in a Dutch out-of-hospital cardiac arrest dispatch system. Resuscitation.

[11] Hansen S.M., Hansen C.M., Folke F., Rajan S., Kragholm K., Ejlskov L. (2017). Bystander defibrillation for out-of-hospital cardiac arrest in public vs residential locations. Resuscitation.

[12] Thepmanee D., Tanaka H., Hiroshi T., Hara T. (2022). Development of smartphone applications for bystander cardiopulmonary resuscitation in the prehospital setting in Thailand. J EMS Med.

[13] Cheng A., Kessler D., Mackinnon R. (2016). Reporting guidelines for health care simulation research: extensions to the CONSORT and STROBE statements. Adv Simul.

[14] Hansen S.M., Hansen C.M., Folke F., Rajan S., Kragholm K., Gerds T.A. (2020). Bystander defibrillation and survival in residential versus public locations. Resuscitation.

[15] Zijlstra J.A., Beesems S.G., De Jong J.S., Tijssen J.G., Koster R.W. (2022). Resuscitation by volunteer responders leads to earlier defibrillation in out-of-hospital cardiac arrest. Resuscitation.

[16] Nabecker S., Nation K., Gilfoyle E., Abelairas-Gomez C., Koota E., Lin Y. (2024). Cognitive aids used in simulated resuscitation: a systematic review. Resusc Plus.

